# Preparatory Switches of Auditory Spatial and Non-Spatial Attention Among Simultaneous Voices

**DOI:** 10.5334/joc.412

**Published:** 2025-01-06

**Authors:** Aureliu Lavric, Elisa Schmied

**Affiliations:** 1 University of Exeter, UK; 2 University of Ulm, DE

**Keywords:** Attention, Cognitive Control, Auditory perception, Auditory processing

## Abstract

Can one shift attention among voices at a cocktail party during a silent pause? Researchers have required participants to attend to one of two simultaneous voices – cued by its gender or location. Switching the target gender or location has resulted in a performance ‘switch cost’ – which was recently shown to reduce with preparation when a gender cue was presented in advance. The current study asks if preparation for a switch is also effective when a voice is selected by location. We displayed a word or image 50/800/1400 ms before the onset of two simultaneous dichotic (male and female) voices to indicate whether participants should classify as odd/even the number spoken by the voice on the left or on the right; in another condition, we used gender cues. Preparation reduced the switch cost in both spatial-and gender-cueing conditions. Performance was better when each voice was heard on the same side as on the preceding trial, suggesting ‘binding’ of non-spatial and spatial voice features – but this did not materially influence the reduction in switch cost with preparation, indicating that preparatory attentional shifts can be effective within a single (task-relevant) dimension. We also asked whether words or pictures are more effective for cueing a voice. Picture cues resulted in better performance than word cues, especially when the interval between the cue and the stimulus was short, suggesting that (presumably phonological) processes involved in the recognition of the word cue interfered with the (near) concurrent encoding of the target voice’s speech.

Seven decades ago Cherry (1953) formulated the *cocktail party problem*: how does a listener “tune in” to one of two or more simultaneous voices and what are the limits of such selective listening? Research conducted since Cherry’s seminal experiments has shone light on multiple aspects of the problem, in particular that: the primary challenge in the cocktail party scenario is not the processing of speech signal masked by other speech containing energy in the same spectral bands (*energetic masking*), but the selection of the relevant spoken message in the face of *informational masking* from the content of the other streams (e.g., Brungart, 2001; Brungart et al., 2001; Darwin, 2008); spatial separability of talkers benefits selection, but voices coming from the same location can also be effectively distinguished based on cues such as fundamental frequency, vocal tract size, prosody, accent, etc. (e.g., Darwin et al., 2003; Darwin & Hukin, 2000); listeners can integrate such physical/perceptual attributes into auditory *objects*, which benefit from the temporal constancy (continuity) of the constituent attributes (e.g., Best et al., 2008; Kitterick et al., 2010; Samson & Johnsrude, 2016); familiarity with a voice improves speech intelligibility (e.g., Holmes et al., 2018a; 2021) – to list only a few key findings relevant to the present study (for reviews, see Bronkhorst, 2015; Shinn-Cunningham & Best, 2015).

A relatively recent development has been the emergence of a body of research concerned with how the listener switches attention among talkers as dictated by the listener’s task goals (e.g., Koch et al., 2011; Koch & Lawo, 2014; Nolden et al., 2019; Monsell et al., 2019; Seibold et al., 2018; Strivens et al., 2024a; Strivens et al., 2024b). This approach has been inspired by *cognitive control of task-set*, which treats attention to a perceptual constituent of a stimulus (such as the target voice in a multi-talker compound) as a component of the listener’s *task set*; other components of the task-set include S-R rules, response effectors, etc. (e.g., Meiran et al., 2000; Monsell, 2015). One of the benefits of applying this framework to attentional switching in the cocktail party setting is that one can adapt experimental paradigms (and associated measures of participant performance) that were developed, extensively tested and optimised to examine switching between task sets (for reviews, see Kiesel et al., 2010; Koch & Kiesel, 2022; Monsell, 2017; Vandierendonck et al., 2010). In the most widely used variant of the task-switching paradigm the participant is first familiarised with two or more simple cognitive tasks whose stimuli typically afford all tasks, and then asked to perform them in an unpredictable sequence; a task *cue* specifies on each trial the task to be performed (Meiran, 1996). The critical contrast of *task-switch* vs. *task-repetition* trials almost invariably reveals longer response times (RTs) and lower accuracy for switches than for repetitions – this performance *switch cost* is thought to have several processing sources (e.g., Elchlepp et al., 2015; Koch & Kiesel, 2022). One key source of the switch cost is the top-down updating/resetting of the task-set parameters required by a task switch – as proposed by the *task-set reconfiguration* account (Rogers & Monsell, 1995; Monsell, 2003; 2017; Vandierendonck et al., 2010). There is substantial, and growing, evidence that at least some task-related perceptual attributes are subject to task-set reconfiguration during a task switch (e.g., Elchlepp et al., 2017; 2021; Kikumoto et al., 2016; Longman et al., 2014; 2016; 2017; Mayr et al., 2013).

A conventional task-switching experiment often involves a change in the relevant perceptual attribute, but it also involves changes in the relevant categorisation, and S-R rules, hence the switch cost may reflect changes in (and reconfiguration of) multiple task-set components. A straightforward adaptation of such a paradigm for investigating specifically switches of attention in the multitalker scenario is to ensure that the target speech stream is the only task component that changes, while keeping the remaining components (particularly the required categorisation and S-R rules) constant. This enables one to isolate the change vs. repetition in the target voice as the only cause of a potential switch cost. Koch and colleagues (Koch et al., 2011) pioneered this approach by developing what will be referred henceforth as the *voice cueing* (or *voice switching*) paradigm: on each trial participants heard via headphones two simultaneous talkers – a man and a woman – presented dichotically (with random gender-to-ear mapping on each trial), each saying a single-digit number. Participants had to categorise the magnitude of the number (as < or > 5) spoken by the voice which was specified by a visual cue indicating the gender of the target voice. The target gender changed unpredictably on half of the trials – which resulted (compared to repetitions of the target gender) in a large switch cost.

The substantial cost of switching the auditory attention to the target voice is consistent with active, time-consuming, top-down, control of attentional shifts, but it is also consistent with ‘passive’ persistence/inertia/priming of the attentional set – which should benefit performance on repetition trials and hinder performance on switch trials. This would be a form of *task-set inertia* (Allport et al., 1994) – passive persistence of tasks-set parameters – widely viewed as one of the sources of the task switch cost (e.g., Monsell, 2003). Indeed, *attentional inertia* for visual attributes has been documented in the task-set control literature. Studies that employed eyetracking or fMRI to examine visual attention in the context of task switching have revealed a tendency to encode the previously (but, on switch trials, no longer) relevant visual dimension (Kikumoto et al., 2016; Longman et al., 2013; Mayr et al., 2013; Yeung et al., 2006) or location (Longman et al., 2014). The task-cueing paradigm provides a way of distinguishing between the contributions of top-down control and task-set inertia: one can manipulate independently the time available for active (top-down) preparation (the cue-stimulus interval, CSI) from the time elapsed from the previous response during which passive inertia dissipates (the response-stimulus interval). Both manipulations have been shown to influence the task switch cost, providing support for both active and passive sources of the performance overhead of switching tasks (e.g., Meiran, 1996).

Of particular relevance to the current study, extending the CSI in the range 0 s to ~1.5 s has tended to result in a substantial reduction in switch cost (the *RISC effect*, Monsell et al., 2006; Van’t Wout et al., 2013; 2015). However, preparation almost never eliminates the switch cost (though see Verbruggen et al., 2007), leaving a non-trivial asymptotic (‘residual’) component. The fact that preparation in advance of the imperative stimulus can have a substantial effect on the task switch cost (unconfounded from the effect of task-set inertia), is consistent with ample evidence of pre-stimulus switch-related EEG activity (for a review, see Karayanidis et al., 2010) and eye-movements (Longman et al., 2014; 2016; 2017; 2021) – both of which are predictive of smaller switch costs (e.g., Elchlepp et al., 2012; Kieffaber & Hetrick, 2005; Lavric et al., 2008; Longman et al., 2014; 2017). These and other kinds of evidence have made the RISC effect one of the clearest correlates of top-down control of task set.

The voice-cueing study by Koch et al. (2011) included a manipulation of the preparation interval which contrasted performance at CSIs of 100 ms and 1000 ms, while keeping the response-stimulus interval constant. They found no reliable evidence that preparation reduced the cost of switching attention between simultaneous voices, although preparation improved the overall performance (collapsing over switches and repetitions). Subsequent studies from the same group that used variants of this paradigm, revisited the effect of preparation on the target voice switch cost (Lawo et al., 2014; Lawo & Koch, 2015; Nolden et al., 2019; Seibold et al., 2018). The outcome was the same – no reliable evidence of such an interaction, except for one experiment in Seibold et al. (2018) where the female voice and male voice alternated as targets in (predictable) runs of two (e.g., m-m-f-f-m-m, etc.) and where the effect of preparation was examined by manipulating the response-stimulus interval. However, because extending the response-stimulus interval provides more time for both preparation (top-down control) and passive dissipation of the attentional set (inertia), one cannot be certain about the contribution of these processes to the observed reduction in switch cost (see above).

The above evidence seemed to suggest that advance task-set reconfiguration may not apply to the auditory attention component of a tasks-set. It is conceivable that shifting one’s auditory attention from one gender (or specific voice) to another in advance of hearing the voices may be difficult/ineffective, possibly because such retuning may require the presence of perceptual input. Such a conclusion would be intriguing in the context of studies that have found preparation to reduce the cost of switching auditory attention (keeping other task-set parameters constant) between different sound dimensions (pitch vs. volume, Nolden & Koch, 2023), or between temporal patterns in sequences of sounds (Nolden & Koch, 2017), as well as studies that examined switching between the auditory and visual modalities (Lukas et al., 2010), and the cost of switching visuospatial attention among different locations (e.g., Logan, 2005). The present study is part of a series of investigations that systematically test the above possibility that the advance task-set reconfiguration account may not apply to selecting in advance a voice in a multitalker setting based on its spatial or non-spatial perceptual attributes.

In a recent study from our laboratory (Monsell et al., 2019) we asked whether preparatory switching of attention to a different voice is indeed limited, as the evidence reviewed above seems to suggest, or whether optimising the conditions for encouraging and detecting preparation may reveal its effect on the switch cost. In task switching, the RISC effect has been reported when switches and repetitions are equiprobable (e.g., Mayr et a., 2013; Monsell & Mizon, 2006; Verbruggen et al., 2007), but it is considerably steeper when switches are less likely than repetitions. Hence, we reduced the probability of a switch of the target voice from 0.5 in the earlier voice-cueing studies to 0.33. We also reduced the proportion of trials where the digits spoken by both voices are mapped onto the same response (*response-congruent* trials) from 50% (in previous studies) to 20%, because only on *response-incongruent* trials (for which the two spoken digits afford different responses) a correct response requires selection of the target voice. In most (though not all, cf., Seibold et al., 2018) previous gender-cueing experiments a participant heard more than one voice of each gender. We reasoned that preparation may be more effective if the listener has to attend to a just one voice of each gender. Furthermore, previous voice-cueing studies used dichotic presentation (with random allocation of genders to ears on each trial). We reasoned that rapid, and presumably unnatural, changes of the (task-irrelevant) voice location (side) may impede/discourage/distract from preparing for the relevant voice identity.^1^ Hence, we used non-localised central (diotic) presentation. Finally, we allowed for the possibilities that preparation may be more effective when the listener is familiar with the voice and/or when the onsets of the two voices are not simultaneous (one lags behind the other). Thus, we compared conditions where the voices were highly familiar (participants’ parents) vs. previously unknown, and where voice onsets were simultaneous vs. successive, whilst also examining the effect of temporal order.

The results obtained by Monsell et al. (2019) confirmed the substantial cost of switching the target voice reported previously. More importantly, the study also found that the large switch cost in the shortest CSI (50 ms) was reduced substantially (~40%) and significantly when the CSI increased to 800 ms. This RISC effect seemed unaffected by familiarity with the voices and their simultaneity or temporal order. In a more recent voice-cueing study (Strivens et al., 2024), which used similar design parameters (diotic presentation of two simultaneous voices, target defined by gender), but also manipulated the probability of a switch of the target voice, we confirmed that preparation can significantly reduce the target voice switch cost, provided the probability of a switch was relatively low (0.25); conversely, preparation did not reduce the switch cost when switch probability was high (0.75). The latter finding suggests that the probability of a switch is a key factor for detecting the effect of preparatory (re)tuning to another voice in the multitalker setting – and that it may in part or largely explain the discrepancy among earlier studies that did not find a significant RISC effect and more recent studies that did.

The voice-cueing studies presented thus far were all focused on selection between simultaneous voices defined by the gender of the talker (presumably based on the associated characteristics of fundamental frequency, vocal tract length, prosodic contour, etc.). In the studies in which only one voice per gender was presented (Monsell et al., 2019; Seibold et al., 2018; Strivens et al., 2024) participants likely learned to tune in to specific talkers. Yet, as Cherry (1953) showed in some of his seminal experiments, the listener is also able to select a voice based on its location/side. Spatial selection of voices in the context of dynamic changes in the location of the target voice was investigated by Best and colleagues (2008), who presented participants with sequences of digits spoken simultaneously from five loudspeakers in front of them. Each sequence consisted of four time steps separated by a delay of 0/250/500/1000 ms. At each step five different voices (one per loudspeaker) each spoke a different number. The target location was cued by a LED on one loudspeaker turning on either for the entire duration of the delay or simultaneously with the onset of the spoken digits – allowing for ample/little/no time for preparation. At the end of each sequence, participants had to report the four numbers presented in the target loudspeaker. The study manipulated the continuity of the target location and the continuity of the voice at the target location (same throughout the sequence vs. random at each step); at non-target locations voices were randomly selected at each step from a larger set of voices. The authors’ primarily aim was to determine whether the effectiveness of auditory tuning improved monotonically with greater continuity of the input – and it did: there was a substantial (and relatively linear) improvement in the accuracy of reporting the digit with every step in the sequence – but only when the target location was constant throughout the sequence. Moreover, this benefit of spatial continuity was greater when the voice at the target location did not change, especially at short delays between the sequence stimuli (see also Best et al., 2010). Best et al. (2008) also found that digit reporting accuracy was substantially worse when the target location changed throughout the sequence than when location was constant. Of particular relevance to the present study, the opportunity for preparation provided by the LED location cue during delays reduced this location switch cost – but only when the voice at the target location stayed the same throughout the sequence.

The research team that developed the voice-cueing paradigm to examine gender-cued selection (e.g., Koch et al., 2011) subsequently investigated spatial attention to voices. In particular, Lawo et al. (2014) cued the target voice by ear/side in some experimental conditions. Their results revealed a large ear/side switch cost – which was not significantly reduced by preparation. Seibold et al. (2018) revisited the effect of preparation on the switch cost, but still found no RISC effect for ear-cued selection, except when the target ear alternated in predictable runs of two (left, left, right, right, etc.). As already noted earlier, the alternating runs design cannot unequivocally distinguish between the effects of active preparation and those of ‘passive’ dissipation of attentional parameters. Other studies compared the effectiveness of gender and location cues. For example, Holmes and colleagues (2018b) modified the classic ‘call sign’ (coordinate response measure) paradigm in which each of several talkers says a sentence of the form “Ready Baron, go to red three now” and the participant responds to the colour-number combination spoken by the talker who says a pre-specified call sign (e.g., “Baron”). Holmes et al. removed the call sign from their speech stimuli (leaving only phrases like “red three now”) and cued the target voice by gender or location, whilst also manipulating the CSI. They found that performance was better for location cues and that it improved with increasing the CSI, but they did not examine the effect of switching the cued feature on performance – the switch cost – or its reduction with preparation.

The above evidence suggests that it may be difficult to shift the auditory attention in advance to a different location (ear), or that this can be done only in some circumstances (cf., Best et al., 2008), thus questioning the applicability of theoretical accounts based on advance task-set reconfiguration (e.g., Monsell, 2003; 2015) to spatial selection in the cocktail party. However, similar considerations regarding non-spatial selection (cued by gender) turned out to be premature (see above). Thus, the primary aim of the present study is to ask: can the listener prepare in advance for a change of the location of voice in the cocktail party setting, as predicted by the advance task-set reconfiguration account? If so, the cost of changing the location of the voice should substantially reduce with preparation. Or, is preparatory spatial selection of a voice limited/ineffective, as suggested by the lack of a significant RISC effect in previous voice-switching studies that used spatial voice cueing?

The second aim of the present study is to investigate whether the target voice switch cost and its reduction with preparation are influenced by trial-to-trial dynamics of a salient task-irrelevant dimension. The previous studies that reported a RISC effect (Monsell et al., 2019; Strivens et al., 2024) used diotic (non-spatial) presentation, hence the only dimension whose features varied over trials was the relevant, non-spatial, dimension related to voice identity. The study by Best et al. (2008) reviewed above found a larger performance cost of switching the location of the target loudspeaker when the voice presented in the target location remained the same throughout the presentation sequence than when the voice in the target location changed during the sequence. Of particular importance to the present study is that Best et al. found a modest (yet significant) reduction in the location switch cost – but only when the voice at the target location remained the same throughout the presentation sequence, suggesting that the transient relationship between features on relevant and irrelevant dimensions may influence the likelihood and/or effectiveness of preparation.

Koch and Lawo (2014) examined in their dichotic gender-cueing paradigm the interaction between the relevant and the irrelevant dimensions and found that the performance switch cost for the relevant dimension (gender) was substantially larger when the location (side) of the cued gender remained the same from one trial to the next than when the location of the cued gender changed. This could arise from to some combination of: (a) a greater benefit of a repetition of the target gender when the location (side) of each gender trial *n* (e.g., male on the left, female on the right) remained the same as on trial *n–1* (male-left, female-right), than when the two genders swapped locations on trial *n* (male-right, female-left), and/or (b) a greater detriment of a target gender switch cost when the locations of the genders were swapped on trial *n* relative to trial *n-1* (as above). The authors interpreted their results in terms of episodic bindings that form between features across the relevant and irrelevant dimensions (e.g., male-left, female-right) – but they did not present a detailed breakdown of the means for the different conditions to ascertain the presence of the patterns (a) and/or (b) above. Of more relevance for the present investigation, there was no manipulation of preparation in Koch and Lawo (2014), so it is not clear how the potential links/bindings between features across the two dimensions may interact with preparation. The above-mentioned study by Holmes et al. (2018b), which also cued participants by gender or location, have examined trial-to-trial dynamics in the mapping between dimensions, which they refer to as ‘configuration’ (male at location x, female at location y) and found better performance when the configuration was the same as on the previous trial than when it changed. However, they did not examine the cost of switching the feature of the relevant dimension and the interaction between preparation and the switch cost and/or the configuration effect.

It is conceivable that the presence of a salient irrelevant dimension that changes unpredictably might altogether discourage participants from engaging in effortful preparation, or considerably reduce its effectiveness – in which case we may find little/no RISC effect. Another possibility, which is in line with the auditory attention being more effective when deployed at the level of multi-dimensional auditory objects (e.g., Best et al., 2008), is that the RISC effect may be present only when the mapping/configuration of features across dimensions (male at location x, female at location y) is kept the same as on trial n-1. We will examine these possibilities both for spatial selection (with ear as the relevant/cued dimension) – in Experiment 1 – and for non-spatial selection (with gender as the cued dimension) – in Experiment 2.

The third aim of the current study concerns the type of the visual cue used to specify the target voice (or location). In its relatively early days, task-switching (task-cueing) research came to the realisation that repetitions of the same cue from one trial to the next are problematic. Comparisons of task repetitions where the cue was repeated with task-repetitions where the cue changed (e.g., Logan & Bundesen, 2003; Monsell & Mizon, 2006), showed that cue repetitions result in a substantial cue encoding benefit (‘cue priming’) thus confounding (inflating) the ‘true’ task-switch cost when there is a single cue per task. This has also been shown in the context of voice-cueing by Koch and colleagues (Koch et al., 2011). To avoid cue repetitions, Monsell et al. (2019) alternated between two semantically transparent visual cues for each gender (voice) – the words “male” and “female” and greyscale silhouette images of a man and a woman (see Figure 1). This enabled the comparison of verbal and pictorial cues – which revealed better overall performance with picture (silhouette) cues. This outcome is somewhat surprising in the context of task switching, where verbal cues have been generally more effective than picture cues even when pictures were highly informative/evocative. For example, Monsell and Mizon (2006) asked participants to switch between identifying a set of coloured canonical shapes by their form or colour. The task cues were the words “colour”/“shape” and two pictures: a collage of the four possible colours and a greyscale collage of the four possible shapes. Despite containing relevant stimulus attributes, picture cues resulted in longer RTs and higher error rates on switch trials than word cues in the short CSI (140 ms) condition. Lavric et al. (2008) used the same tasks and cues and found longer RTs and higher error rates for picture cues even at a long CSI (800 ms). It may be premature to draw the conclusion that words are less effective voice cues than pictures based on a single study (Monsell et al., 2019). Hence, in the present study we revisit the comparison between verbal and pictorial cues both when the target voice is defined by gender and when it is defined by location (ear). Based on the task-switching literature, one might predict that word cues may result in more effective activation of the relevant attentional set and therefore better overall performance and possibly a smaller switch cost (cf. Monsell & Mizon, 2006). However, based on the voice-switching study by Monsell et al. (2019) one would predict the opposite – that picture cues may result in superior performance and possibly smaller switch cost.

**Figure 1:**
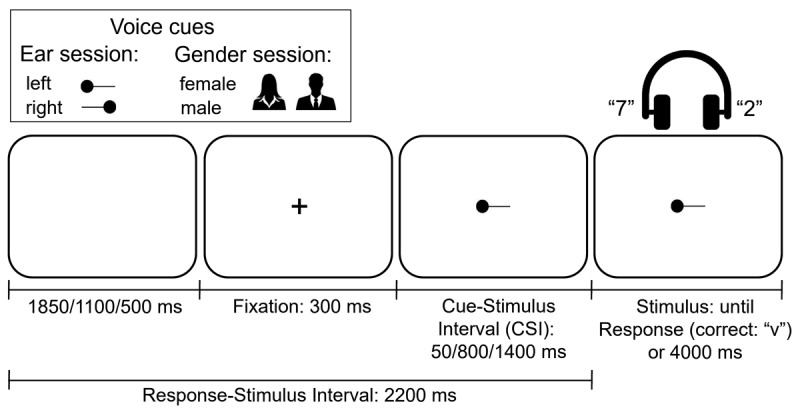
The Cues Used in the Two Experimental Sessions and the Temporal Structure of a Trial (Illustrated for the Ear Session).

## Experiment 1

Our first experiment consisted of two testing sessions, each with its objective. In one session (henceforth the ‘ear session’), we presented dichotically the voices of two speakers – a female and a male – each saying a single-digit number. We instructed participants to attend to their left or right ear (side) and specified the target side on each trial using a visual cue. Crucially, we varied the cue-(voice) stimulus interval (CSI) available for preparation and an examined the effect of this manipulation on the switch cost. In order to encourage preparation and maximise the chances of detecting it, we implemented parameters of the experimental paradigm that revealed a RISC effect for gender-cued selection (see Introduction): a single voice per gender, a relatively low probability of a switch of the target ear/side (0.33), and a high proportion of response-congruent trials (80%). The talker presented to the target ear changed randomly, hence we also investigated whether trial-to-trial changes in gender-ear mappings (e.g., female in ear x, male in ear y) had an effect on the switch cost and its reduction with preparation – the RISC effect.

When Experiment 1 was conducted, there had been only one experiment which reported a RISC effect for gender-cued unfamiliar simultaneously presented voices (Monsell et al., 2019, Experiment 2). To confirm this finding, in another session (henceforth: ‘gender session’) we conducted a direct replication of Monsell et al.’s (2019) experiment, using the same design, procedure and materials, except for a different set of voices. Although the RISC effect for gender-cued voices has since been confirmed in our more recent study (Strivens et al., 2024), there are at least two reasons for briefly presenting the results from the ‘gender session’. First, there is value in further replication. Second, perhaps more importantly, the presentation of the comparison between verbal and pictorial cues for both ear cueing and gender cueing allows for a comprehensive examination of the advantage reported in Monsell et al. (2019) of pictures over words as voice cues in the cocktail party setting (see Introduction).

### Method

#### Data Accessibility Statement

Materials, scripts and data are available at https://osf.io/g72mk/.

#### Participants

Thirty-nine participants were recruited on the University of Exeter campus and paid a flat rate of £14 plus a performance-related bonus of £1.00–£3.60 (see below for details on the bonus) for taking part in the two 45–50min sessions of the study whose procedure had been approved by the local ethics committee (Department of Psychology, University of Exeter). Since the experiment’s paradigm required participants to comprehend spoken English in relatively challenging conditions of energetic and informational masking from a simultaneous speech stream, participants were required to have native or native-level proficiency in English. Three participants were deemed outliers and their data was excluded from analyses (and subsequently replaced): two had very high error rates in one of the two sessions (>3 SDs above the mean of the sample), and one had both a very high error rate and a very long mean RT (both ~3 SDs above the mean) in one session. This resulted in the target sample of 36 subjected to statistical analyses (23 females, 13 males; mean age = 24, SD = 4.84).

The target sample of 36 was determined based on both statistical power considerations and the necessary balancing over participants of key aspects of the experimental design. With regard to statistical power, this sample size is more than adequate based on the following three criteria. First, our recent *a priory* analysis using G*Power (Faul et al., 2007) based on effect sizes in 10 task-cueing experiments conducted in our laboratory over the last decade – all of which examined the critical switch/repeat × CSI interaction (testing the RISC effect) – yielded, for observing the range of effect sizes .313 ≤ ηp^2^ ≤ .815 observed in our previous experiments, for α ≤ .05, a mean sample size estimate of N = 9.7, with a median = 10 and a range of 5–14, required to achieve the power of 0.8; and an N = 11.9 (median = 12, range: 6–12) required to achieve power 0.9 (for further details, see Monsell et al., 2019). The present N = 36 is at least three times larger than these a priori estimates. Second, based on simulations using data acquired in a mega-study, Brysbaert and Stevens’ (2018), concluded that a well-powered within-participants experiment requires a minimum of 1600 observations (over participants and trials) in the smallest cell of interest to detect a smaller than medium-sized effect. In the current experiment, the smallest cell of interest (the switch/repeat × CSI interaction) consisted of ~47 trials per participant in each of the two sessions if one takes into account the exclusion of some trials from analyses (see below for details), resulting in a total of ~1692 observations per session over the 36 participants – which meets the recommended 1600 observations even if one accounts for a further attrition of ~5% observations, such as the exclusion of errors in RT analyses. Third, the present sample is 50% larger than the sample of our previous study (Monsell et al., 2019), which found a large (ηp^2^ = .387) and highly significant RISC effect, and on whose paradigm served the present experimental design is modelled.

Importantly, the n = 36 also allowed perfect balancing of two aspects of the experiment over participants: the order of the blocks of different preparation intervals (CSIs) – six different block orders were used, and the six female-male pairs of voices (only one of which was head by a participant in a given session, see below for further details). There were 36 combinations of these variables.

#### Design, Task, Apparatus, Materials and Procedure

Both sessions of the experiment were conducted in a sound-attenuated laboratory booth (with the experimenter outside) using E-Prime 2 (Psychology Software Tools). The participant sat in front of a 19” monitor connected to a PC running Windows 7. The auditory (speech) stimuli were presented via Sennheiser DT 770 PRO 250 OHM headphones (Sennheiser Electronic) connected to the PC. In each session, participants were presented with compounds of one female voice and one a male voice (neither known to the participant before the experiment), each saying a number between 2 and 9. The task was always to attend (listen) to one of the voices and classify the number spoken by the target voice as odd vs. even. In one session, the target voice was defined by the ear/side (left vs. right) where the participants heard the voice, whereas in the other session the target voice was defined by the gender of the speaker. The two sessions ran on different days (at an interval of minimum two days and maximum 14 days). The order of the two sessions was balanced over participants independently of the balancing of CSI and voice pair (see above and below).

Six male and six female native speakers of English aged 20–29 were recorded in a sound-attenuated booth speaking the numbers 2–9 ten times – these were subsequently segmented into separate utterances for each number, selected and edited to remove non-speech periods at the beginning and the end and to ensure similar sound amplitude, and paired to create six female-male voice pairs. Each participant was exposed to two voice pairs, one pair per session. In the ear session the presentation of voices was lateralised (dichotic), whereas in the gender session the two voices were presented to both ears (diotically). The six voice pairs were equally represented over participants and sessions by rotating them over sub-groups of six participants: participants 1, 7, 13, 19, 25 and 31 heard voice pair 1 in the gender session and voice pair 6 in the ear session; participants 2, 8, 14, 20, 26 and 32 heard voice pair 2 in the gender session and voice pair 5 in the ear session; participants 3, 9, 15, 21, 27 and 33 heard voice pair 3 in the gender session and voice pair 4 in the ear session, etc.

##### Ear Session

On each trial the side (ear) where the target voice would be heard was specified by one of four greyscale cues – the words “left” (15 × 8 mm) and “right” (21 × 8 mm; 21 × 11 mm including the lower part of “g”)^2^ displayed in lower case Arial font, and the 27 mm × 11 mm arrow-like picture cues shown in Figure 1; the participant’s eyes were ~50 cm from the display. To unconfound voice switches from cue switches (see Introduction), the cue was never repeated, even when the target voice was repeated; this was achieved by alternating word and picture cues. The opportunity for preparation was manipulated by varying the interval between the cue and the voice compound; there were three such cue-stimulus intervals (CSIs): 50 ms, 800 ms and 1400 ms (see Figure 1). The cue was preceded by a 300 ms fixation cross (+). To unconfound preparation from the potential “passive” dissipation/decay of the attentional settings from the preceding trial, the response-stimulus interval (RSI) was kept constant at 2200 ms by varying the duration of a blank display that followed the participant’s keypress (and preceded the fixation on the following trial): 1850 ms, 1100 ms and 500 ms (corresponding to the increasing CSI). The CSI (and the corresponding RSI) was constant within a block of 61 trials, but varied over the 12 blocks in six possible orders (block sequences) balanced over participants – each sequence consisted of repeating four times one of the six permutations of three CSI values (for example, one CSI order over the 12 blocks was: 800, 50, 1400, 800, 50, 1400, 800, 50, 1400, 800, 50, 1400).

The main part of the experiment consisted of 12 blocks of 61 trials – 60 analysable trials plus a start-up trial unclassifiable as a switch/repeat of the target voice and therefore excluded from analyses. Of the total of 720 trials (excluding the start-up trials), 80% were response-incongruent – where the numbers spoken by the man and the woman required different responses. The remaining 20% trials were response-congruent (the numbers spoken by the two voices required the same response). As in our recent voice-cueing studies (Monsell et al., 2019; Strivens et al., 2024, Exp. 1), congruent trials were not subjected to analysis, because on these trials a correct response did not require the target voice to be selected – we included these in the experiment to discourage the strategy of always listening to one voice (e.g., the female) and making the opposite response when the other voice was cued.^3^ The target ear/side switched unpredictably with a probability of 0.33. This resulted in 64 response-incongruent analysable switch trials in each CSI. We excluded trials where any of the two voices repeated the number it said on preceding trial or where the number spoken by the target voice was the same as on the previous trial (whether it was the same voice saying it or not). This resulted in ~47 trials in the smallest cell of interest (incongruent switch trials for each CSI).

On the first trial of each block, the cue (word or picture) was picked randomly and then the cue alternated between word and picture for the remainder of the block. Participants were instructed to press the “v” key on the computer keyboard with the left index finger if the number was odd and the “m” key with the right index finger if the number was even. The response deadline was 4s. If the participant made an error or did not respond before the deadline the messages “ERROR” or “NO RESPONSE” were displayed for 2s. Participants were told that the to-be-attended side would change from one trial to the next unpredictably on one third of the trials and instructed to “respond as quickly as possible while avoiding errors”. After each block, they saw their mean correct RT and error rate, and a performance score (computed as RT/10 + 5 × number of errors). They received a “bonus point” worth £0.30 when the score was lower than their previous average score for the blocks of the same CSI. After being notified of the performance score and bonus point, the participant was given the opportunity to take a short self-paced break before they started the next block. If the number of bonus points accumulated at the end of a session was ≤3, the participant was paid a minimum bonus of £1.

Unique randomised trial sequences were generated for the main part of the session for each participant using custom-written code subject to the following constraints. For the trials where the target ear/side was repeated, the 32 different incongruent number pairs spoken by the two voices were represented equally within each participant over all the combinations of the design factors target ear (left/right) × CSI × ear switch/repeat × voice (male/female), but not voice cue – which was randomised. For the trials on which the target ear switched, the 32 different incongruent number pairs were represented equally over three design factors within each participant: target ear (left/right) × CSI × ear switch/repeat, but some combinations of these three variables were twice as likely when the female voice was on the cued side than when the male voice was on the cued side – however this relative imbalance was controlled over pairs of participants (it was reversed in even-numbered participants relative to odd-numbered participants). For the relatively rare (20%) response-congruent trials that were target ear repetitions, the eight possible numbers heard in the target ear were equally represented over CSI × switch/repeat × voice (male/female), with the number heard in the non-target ear selected randomly among the four available values. Finally, for the response-congruent trials that were target ear switches, both numbers (the one heard in the target ear and the one heard in the non-target ear) were selected randomly.

Before the main part of the experiment, each participant completed two practice phases. During the first practice phase, whose aim was to familiarise the participants with voices, cues and the number categorisation, and provide the participant with the opportunity to adjust the volume of the auditory input (thus allowing for individual differences in auditory acuity), participants heard one voice at a time saying a number that had to be categorised by parity. As in the main part of the experiment, the auditory (voice) stimulus was preceded by a blank display, followed by a fixation cross, followed by one of the two voice cues (word or picture). Participants heard each voice saying each number twice, once with each cue, once on the left and once on the right, resulting in 32 trials in a random sequence, over which the different cues, and combinations of voices and ears, were equally represented. The procedure of the second practice phase was equivalent to the procedure in the main part of the experiment (see above), except that: it consisted of shorter and fewer blocks (3 × 24 trials each), and CSI order was fixed across practice blocks from the longest to the shortest CSI (1400 ms, 800 ms, 50 ms), to help participants adjust to the voice cue becoming necessary (in the first practice phase, where a single voice per trial was presented, the cue was redundant).

##### Gender Session

The gender session was a direct replication of Experiment 2 from Monsell et al. (2019) – hence it had the same design and used the same materials except for a different set of voice stimuli (see above). Most aspects of this session were equivalent to the ear session above except for the following. The criterion for selecting the target voice was its gender, rather than the side/ear where the voice was heard. The obvious design implication was that the independent variable switch/repeat now referred to a change/repetition in the voice gender (and its identity, because there was a single voice per gender).^4^ As in the ear session, the switches of gender were unpredictable and less likely than repetitions, occurring on exactly 1/3 of the trials. The voices and voice pairs were the same as in the gender session, but their presentation was diotic/central (as in Monsell et al., 2019) rather than dichotic. The word and picture cues specifying the target voice gender were “female” or “male” in Arial font, 34mm × 8mm and 24mm × 8mm, respectively, and the black silhouette of a woman (27 mm × 29 mm) or a man (32 mm × 29 mm), see Figure 1.

The unique randomised trial sequences generated for the main part of the session for each participant ensured that the 32 different incongruent number pairs spoken by the two voices were represented equally over all the combinations of the design factors (except cue type, which was randomised): target voice gender (male/female) × CSI × switch/repeat. For the (relatively rare) congruent number pairs, the eight possible numbers spoken by the relevant voice were used equally in the combinations of all design factors (except cue), whereas the number spoken by the irrelevant voice was sampled randomly among the set of four possible even or four possible odd values.

The two practice phases in the gender session were equivalent to those in the ear session, except for the diotic (rather than dichotic) presentation of the voice cues which specified the gender rather than the ear/side.

### Results

From the 47 (on average) trials available for the smallest cell of analysis (switch trials for each CSI) after excluding the infrequent response-congruent trials (included only to prevent strategic listening to just one voice) and repetitions of spoken numbers (see Method), we further discarded from RT and error analyses 6.8% of trials consisting of the first trial of each block and trials following errors (whose switch/repeat status is indeterminate). In addition, we excluded from RT analyses trials containing errors (7.7%) – incorrect keypresses or failures to respond before the deadline. Statistical analyses (ANOVAs) were conducted using SPSS, version 28 (IBM SPSS Statistics). The correction for violations of sphericity (Huynh-Feldt) was applied where necessary, but uncorrected degrees of freedom are reported. The relevant descriptives are presented in the figures. The breakdown of mean RTs and error rates by all the combinations of ANOVA factors can be found online (see link above).

#### Ear Session: the Effect of Preparation on the Switch Cost

**Figure 2:**
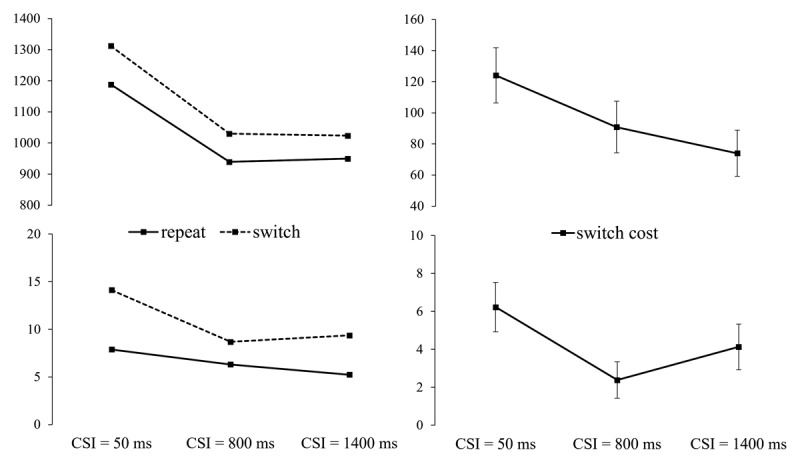
Left: RT and Errors for the Ear Session, as a Function of Cued Ear (Side) Switch vs. Repeat and CSI. Right: RT and Error Switch Costs (with SEs) as a function of CSI.^5^

Switch/repeat (of the cued ear/side) × CSI × cue (word/picture) ANOVAs revealed a significant main effect of Switch/repeat (switch cost) for both RTs, *F*(1,35) = 61.85, *p* < .001, ηp^2^ = .639, and errors, *F*(1,35) = 23.70, *p* < .001, ηp^2^ = .404, and a significant main effect of CSI (RTs, *F*(2,70) = 140.66, *p* < .001, ηp^2^ = .800; errors, *F*(2,70) = 15.82, *p* < .001, ηp^2^ = .311), reflecting an overall monotonic benefit of a longer CSI. The key interaction between switch/repeat and CSI was significant for both RTs, *F*(2,70) = 3.61, *p* = .032, ηp^2^ = .093, and errors, *F*(2,70) = 4.18, *p* = .026, ηp^2^ = .107, revealing a RISC effect for both measures (see Figure 2). Despite the substantial RISC effect (40% for RTs and 62% for the errors), the switch cost at its minimum (at CSI = 1400 ms for RTs and at CSI = 800 ms for errors) was substantial. For RTs the main effect of switch/repeat in the ANOVA for CSI = 1400 ms was significant, *F*(1,35) = 24.82, *p* < .001, ηp^2^ = .415. For the error, the switch cost at CSI = 800 ms for the errors was also significant, *F*(1,35) = 6.14, *p* = .018, ηp^2^ = .149. Thus, preparation substantially reduced, but did not eliminate, the switch cost for both RT and errors, suggesting a non-trivial asymptotic (‘residual’) switch cost.

The ANOVAs which included all CSIs also revealed a significant main effect of cue, for both RTs, *F*(1,35) = 28.66, *p* < .001, ηp^2^ = .450, and errors, *F*(1,35) = 14.14, *p* < .001, ηp^2^ = .288, reflecting 59 ± 11 ms faster responses and a 2.62 ± 0.7% lower error rate with picture cues (1044 ms; 7.29%) than with word cues (1103 ms; 9.91%). For errors, cue interacted significantly with switch/repeat, *F*(1,35) = 4.95, *p* = .033, ηp^2^ = .124, which reflected a larger switch cost on word cue trials than on arrow-like cue trials (5.53 ± 1% vs. 2.96 ± 1.09%). For RTs, the significant cue × CSI interaction, *F*(2,70) = 37.21, *p* < .001, ηp^2^ = .515, reflected a reduction in the performance handicap for the word cue trials (154 ms, 25 ms, –2 ms) as CSI (and presumably time available for cue encoding) increased. The cue × switch/repeat × CSI interaction did not approach significance for either RTs or errors (both Fs < 1, ns.).

#### Ear Session: Interactions between Voice Dimensions

Given the dichotic presentation of the two voices in the ear session (and the random allocation of the male/female speakers to ears over trials), there was a task-irrelevant, non-spatial, dimension (male voice vs. female voice on the side specified by the cue). To examine the possible role of the mappings between features on the relevant dimension and features on the relevant dimension (e.g. male-left, female-right), in particular, whether hearing each voice on the same side as on preceding trial influenced the performance switch cost for the relevant dimension and its reduction with preparation, we conducted repeated-measures ANOVAs with the factors switch/repeat (switch vs. repetition of the feature on the relevant dimension – ear), MappingTransition (same as on trial n-1 vs. different) and CSI; to avoid repetition, we only report here effects involving the MappingTransition factor.

**Figure 3:**
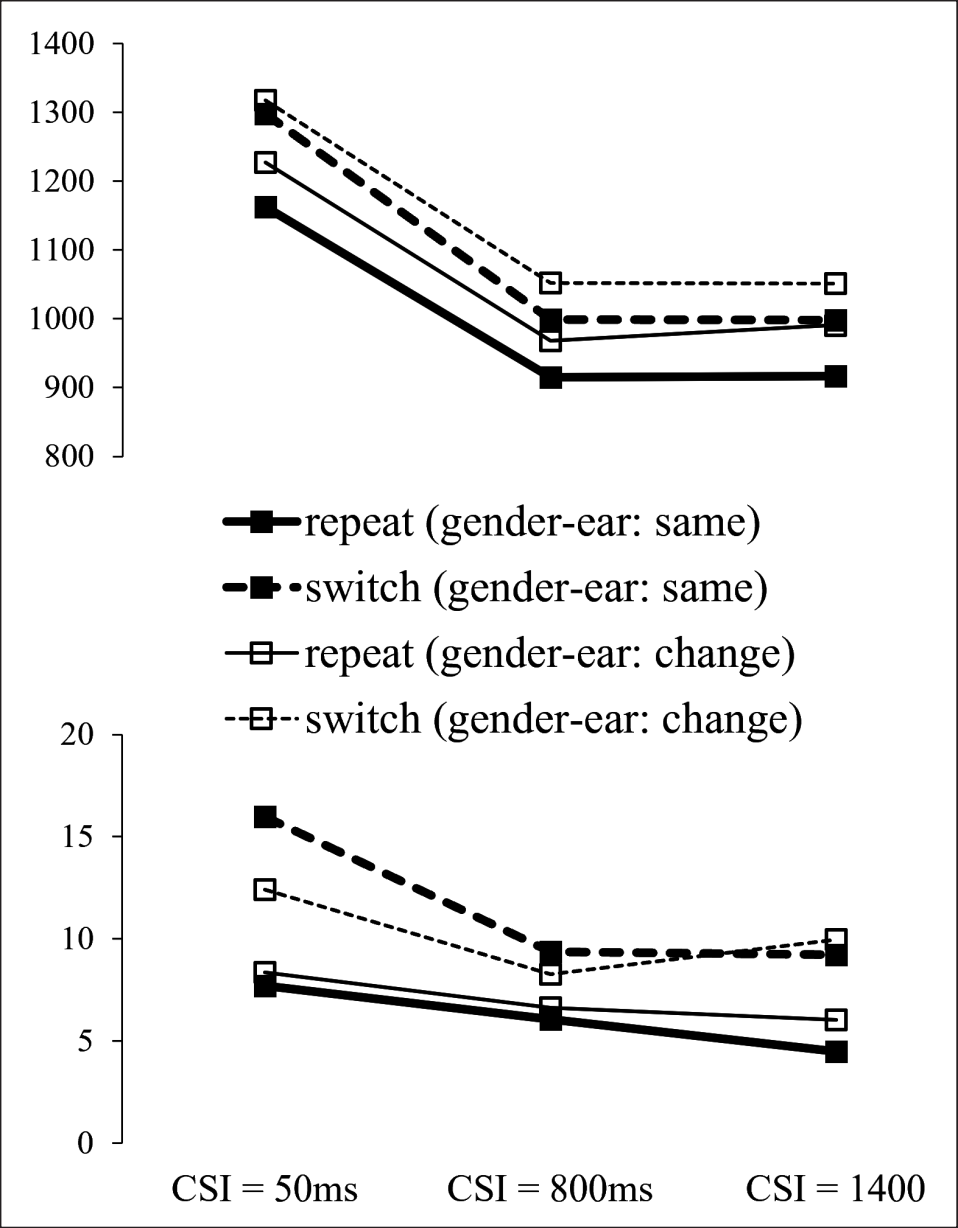
RT and Error Rate for the Ear Session, as a Function of Cued Ear (Side) Switch vs. Repeat, CSI and Gender-Ear Mapping Transition (the Same or Changed Relative to Trial n–1).

The main effect of MappingTransition was significant for RTs, *F*(1,35) = 32.82, *p* < .001, ηp^2^ = .484, reflecting faster responses when gender-ear mappings were repeated from one trial to the next than when they were reversed (see Figure 3), but not for errors, *F*(1,35) = 0.13, *p* = .702, ηp^2^ = .004. Although numerically the RT effect of MappingTransition seems to be larger for ear repetitions than for ear switches, its interaction with Switch/repeat was not significant, *F*(1,35) = 3.02, *p* = .091, ηp^2^ = .079, nor was its interaction with CSI, *F*(2,70) = 0.67, *p* = .507, ηp^2^ = .019, the latter reflecting a comparable benefit over CSIs of encountering the same mappings as on trial n-1. Importantly, there was no evidence to suggest that the effect of preparation on the switch cost (the RISC effect) was influenced by having the same gender-ear mappings as on trial n-1 or not, as indicated by the 3-way interaction MappingTransition × switch/repeat × CSI that did not approach significance, *F < 1*, ns.

#### Gender Session

**Figure 4:**
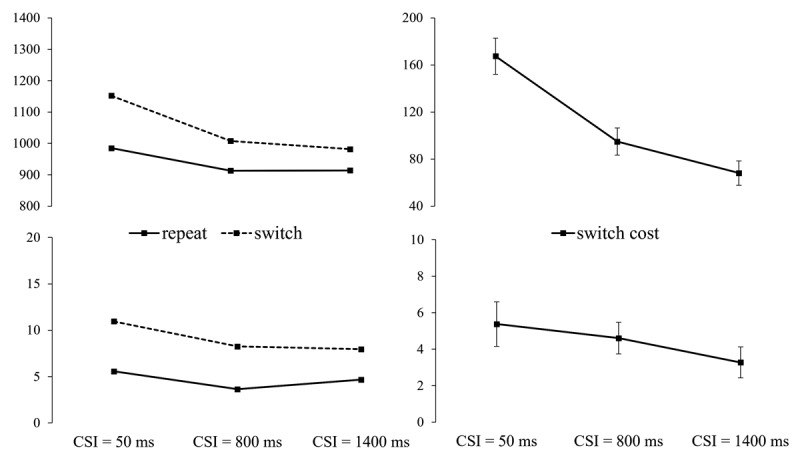
Left: RT (in ms) and Error Rate (%) from Experiment 1, Gender Session, as a Function of Cued Gender Switch vs. Repeat and CSI. Right: RT and Error Switch Costs (± SE) by CSI.

Repeated measures ANOVAs with the factors switch/repeat (of the cued gender/talker), CSI and cue (word/picture) revealed substantial and highly-significant switch costs (main effects of switch/repeat) for both RTs, *F*(1,35) = 122.19, *p* < .001, ηp^2^ = .777, and errors, *F*(1,35) = 48.73, *p* < .001, ηp^2^ = .582. The main effect of CSI was also significant (RT, *F*(2,70) = 38.48, *p* < .001, ηp^2^ = .524; errors, *F*(2,70) = 6.20, *p* = .003, ηp^2^ = .151), reflecting generally better performance as CSI increased. As one can see in Figure 4, preparation (a longer CSI) reduced the switch cost for both RTs and errors. For RTs this RISC effect was significant, as indicated by the switch/repeat × CSI interaction, *F*(2,70) = 28.81, *p* < .001, ηp^2^ = .452; for error rates this interaction did not approach significance, *F*(2,70) = 1.29, *p* = .282, ηp^2^ = .035. As one can see, the RISC effect is steep between CSIs of 50 ms and 800 ms, after which it decelerates towards the 1400 ms CSI. As in the ear session, we tested the significance of the switch cost at its minimum (as an estimate of the asymptotic/residual switch cost) for RTs – where CSI significantly reduced the switch cost. The switch/repeat × cue ANOVA for CSI = 1400 found a significant main effect of switch/repeat, *F*(1,35) = 42,42, *p* < .001, ηp^2^ = .548, indicating that, despite being reduced by 59% from CSI = 50 ms to CSI = 1400 ms, the switch cost remained a substantial at (or near) its asymptote.

There was also a significant main effect of cue (RT, *F*(1,35) = 42.13, *p* < .001, ηp^2^ = .546; errors, *F*(1,35) = 6.86, *p* = .013, ηp^2^ = .164), reflecting 42 ± 7 ms faster responses and a 1.64 ± 0.63% lower error rate with picture cues (971 ms; 6.02%) than with word cues (1013 ms; 7.66%). For RTs, the cue interacted significantly with CSI, *F*(2,70) = 19.82, *p* < .001, ηp^2^ = .362, reflecting a monotonic reduction in the difference between the cues (91 ms, 26 ms, 11 ms) as CSI increased. The interactions between cue and switch/repeat, and cue, switch/repeat and CSI were not significant for either RTs, *F*(1,35) = 2.45, *p* = .127, ηp^2^ = .065; *F*(2,70) = .92, *p* = .388, ηp^2^ = .026; or errors, *F*(1,35) = 2.67, *p* = .111, ηp^2^ = .071; *F*(2,70) = 1.77, *p* = .179, ηp^2^ = .048.

### Discussion

The primary aim of Experiment 1 was to determine if preparatory spatial tuning of auditory attention to a voice in a cocktail party setting can reduce the performance cost of switching the location (side) of the target voice. The ear session revealed such an effect – the RISC effect was significant for both RTs and errors. Moreover, the RISC effect did not significantly interact with trial-to-trial repetition/change in the gender/voice-ear mappings (male voice in ear x, female voice in ear y) suggesting effective intra-dimensional shifts of auditory attention in advance of the stimulus compound. This is not to say that the task-irrelevant (gender) dimension did not influence performance at all. Hearing the same voice in the same ear as on trial n-1 (maintaining the same ear-gender mapping) led to better performance both when the cued side repeated and when it switched; this benefit was numerically (but non-significantly) larger for target side repetitions. This effect is consistent both with the effect reported by Koch and Lawo (2014) for gender-cueing,^6^ and with Best et al.’s (2008) finding of a greater location repetition benefit when the voice presented at that location remained the same throughout a presentation sequence (see Introduction). Our findings support the proposals by the authors of these studies that features may become linked/bound across dimensions (possibly resulting in ‘auditory objects’). However, top-down preparatory switching of spatial attention (as indexed by the RISC effect) seems to be relatively unaffected by such cross-dimension bindings. The gender session replicated our earlier findings (Monsell et al., 2019; Strivens et al., 2024) that when participants are asked to select between two simultaneous diotic voices, non-spatial cueing the target voice in advance of the stimulus also results in a substantial and statistically significant RISC effect.

In both sessions picture cues (silhouettes and arrow-like images) resulted in better performance and, for the error rate in the ear session – a smaller switch cost – than verbal cues. This result not only confirms the finding from Monsell et al. (2019), but also extends it to spatial cueing of the target voice. We will revisit the difference between the two types of cue in Experiment 2 and in the General Discussion.

However, one ‘piece of the jigsaw’ is still missing. We have demonstrated (in three studies, including the present gender session) that extending the preparation interval reduces the cost of switching the target voice gender by 40–60% – but in all these studies voices were presented diotically/centrally, hence neither voice could be perceived by the participant as having a location. On the other hand, in the study by Koch et al. (2011) and all the other subsequent voice-cueing studies where RISC effects were generally non-significant (see above) the presentation was dichotic (spatially lateralised). It is therefore important to ascertain that the substantial RISC effect for gender-cued selection found in the gender session and previously generalises to dichotic presentation – which introduces the task-irrelevant ear/side dimension. The presence of the irrelevant (spatial) dimension also allows us to examine for non-spatial (gender cued) selection the effects of the trial-to-trial dynamics of gender-ear mappings – as we have done in the ear session in Experiment 1.

## Experiment 2

The aim of Experiment 2 was to determine if the index of preparatory switching of attention from one gender (speaker) to another – the RISC effect documented in our recent studies (see Introduction) and confirmed in the gender session of Experiment 1 – will still be present when the task-irrelevant ear (side) changes unpredictably. The paradigm combines aspects of the paradigms used in the two sessions of Experiment 1 (gender cueing but with dichotic presentation) and uses the parameters found to be conducive to (and/or optimise the detection of) preparation effects in our previous experiments: a relatively low switch probability (0.33), a single voice per gender and a low proportion of response-congruent trials (20%). The use of the same verbal and pictorial cues as in Experiment 1 (gender session) will allow us to compare again their effectiveness in conditions of dichotic presentation.

### Participants

Thirty-eight participants from the Exeter University campus with native-level proficiency in English, took part in the single 45min session of the experiment in exchange for course credits. In addition, participants were paid the same performance-related bonus (£1.00–£3.60) as in Experiment 1. The procedure of the experiment was approved by the local ethics committee (Department of Psychology, University of Exeter). The data obtained from two participants were excluded from the analysis: one participant had an error rate >3 SDs over the mean of the sample; the second participant appeared to press the response keys at random in the final four testing blocks (1/3 of the trials). The target sample of 36 participants included nine males and 27 females, with the mean age of 20.53 (SD = 1.13). Because the design of the present experiment is closely modelled on the gender session in Experiment 1, all the considerations regarding statistical power presented there also apply here. We also note that the effect size of the critical interaction between switch/repeat and CSI in Experiment 1 was large (ηp^2^ = .452) and well within the range of effect sizes for this interaction in the experiments on which our a-priori power analysis was based (.313 ≤ ηp^2^ ≤ .815, see Exp. 1, Method).

### Design, Task, Apparatus, Materials and Procedure

The design and materials (including the voice stimuli and cues) were the same as in the gender session in Experiment 1, except the following two aspects. First, voices were presented dichotically (as in the ear session in Experiment 1), rather than diotically. Second, the automatic generation of unique trial sequences for each participant had to be adapted to take into account the extra design variable – ear/side of the target gender. For the trials where the target gender (voice) was repeated, the 32 different incongruent number pairs spoken by the two voices were represented equally over all the combinations of the design factors: target gender (female/male) × CSI × voice switch/repeat × ear (left/right), but not voice cue which was randomised. For the target gender switch trials, the 32 different incongruent number pairs were represented equally over three design factors within each participant: target gender (female/male) × CSI × voice switch/repeat, but some of those combinations were twice as likely when the target gender was presented to one ear than when the target gender was presented to the other ear – however this relative imbalance was controlled over pairs of participants, by reversing in even-numbered participants relative to odd-numbered participants. For the (relatively rare, 20%) response-congruent trials that were target gender repetitions, the eight possible numbers spoken by the target gender were equally represented over target gender (female/male) × CSI × switch/repeat × ear (left/right), with the number spoken by the non-target gender selected randomly among the four available values. Finally, for the response-congruent trials that were target gender switches, the numbers spoken by both voices were selected randomly.

### Results

The trial exclusion criteria were the same as in Experiment 1 and the proportions of excluded trials were essentially the same. As with the ear session from Experiment 1, we first present the analysis of the switch cost and its reduction with preparation and then follow it with the analysis of the interaction between the gender and ear dimensions. As in Experiment 1, relevant descriptives are presented in figures, and the breakdown of mean RTs and error rates by all the combinations of ANOVA factors can be found online.

#### The Effect of Preparation on the Switch Cost (see Figure 5)

**Figure 5:**
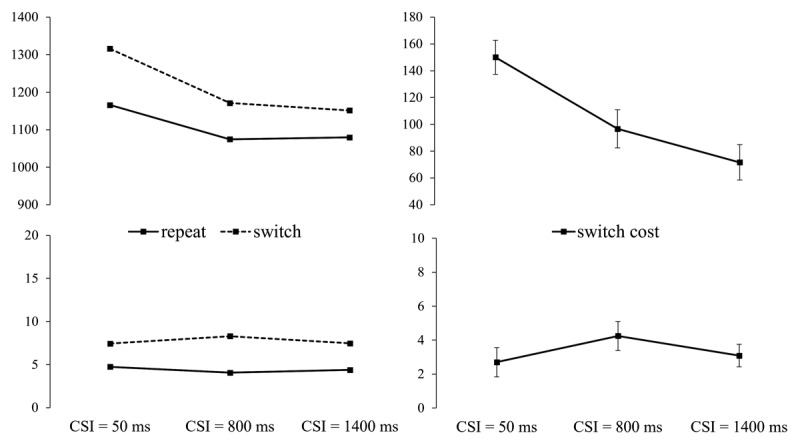
Left: RT and Error Rate from Experiment 2 as a Function of Cued Gender Switch vs. Repeat and CSI. Right: RT and Error Switch Costs (with SEs) by CSI.

The (gender) switch/repeat × CSI × cue ANOVAs found the switch cost (main effect of switch/repeat) to be significant for both RTs, *F*(1,35) = 126.52, *p* < .001, ηp^2^ = .783, and errors, *F*(1,35) = 59.95, *p* < .001, ηp^2^ = .631. There was also a significant main effect of CSI for RTs, *F*(1,35) = 5.68, *p* = .023, ηp^2^ = .140, reflecting faster responses overall when CSI increased. Importantly, the switch cost reduced substantially (52%), and significantly, for RTs, *F*(2,70) = 11.80, *p* < .001, ηp^2^ = .252, but not for errors, *F*(2,70) = 0.95, *p* = .392, ηp^2^ = .026. Despite its steep reduction with preparation, the switch cost remained significant at its minimum as indicated by the switch/repeat × cue ANOVA for CSI = 1400 ms, *F*(1,35) = 29.58, *p* < .001, ηp^2^ = .458.

As in Experiment 1, picture cues resulted in 20 ± 8 ms shorter RTs than word cues, *F*(1,35) = 5.68, *p* = .023, ηp^2^ = .14. CSI and cue interacted significantly for RTs, *F*(2,70) = 5.09, *p* = .009, ηp^2^ = .127, where the performance advantage for picture cues over word cues was largest for CSI = 50 ms, reducing in CSI = 800 and altogether disappearing in CSI = 1400 (46 ms, 21 ms, –7 ms, respectively). There was also a significant cue × CSI interaction for errors, *F*(2,70) = 6.88, *p* = .002, ηp^2^ = 0.164, reflecting better performance with picture cues (5.01%; 6.99%; 6.01%) than with word cues (7.17%; 5.37%; 5.48%) at the shortest CSI, and a reversal of this difference at the two longer CSIs. The interactions between cue and switch/repeat, and cue, switch/repeat and CSI did not approach significance for either RTs, *F*(1,35) = .02, *p* = .889, ηp^2^ = .001; *F*(2,70) = 2.92, *p* = .061, ηp^2^ = .077; or errors, *F*(1,35) = .41, *p* = .528, ηp^2^ = .012; *F*(2,70) = .68, *p* = .508, ηp^2^ = .019.

#### Interactions between Voice Dimensions (see Figure 6)

**Figure 6:**
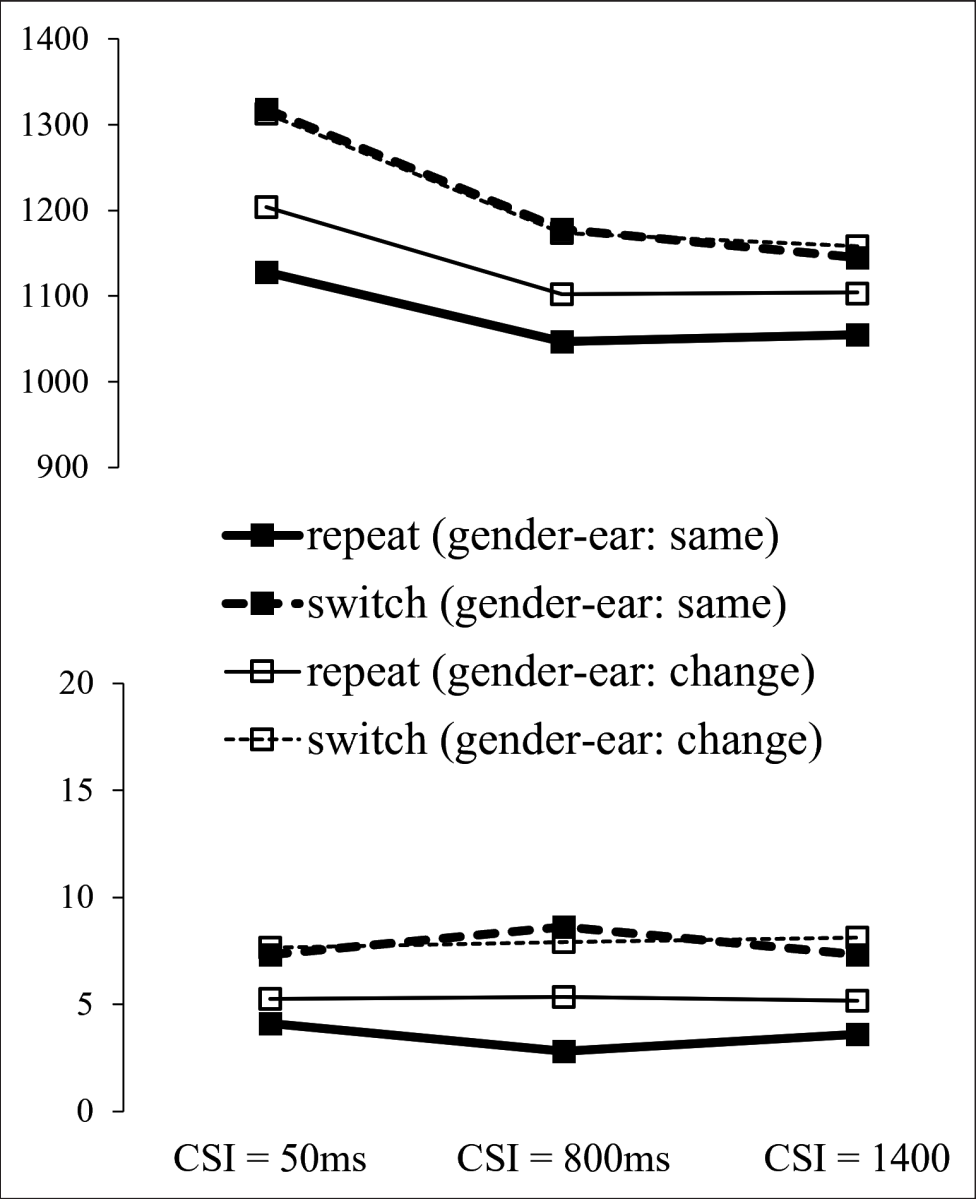
RT and Error Rate from Experiment 2 by Cued Gender Switch vs. Repeat, CSI and the Gender-Ear Mapping Transition.

As in the ear session of Experiment 1, we examined the potential role of bindings across dimensions and whether hearing each voice on the same side as on trial n-1 or on the other side influences the performance switch cost for gender and its reduction with preparation. To avoid duplication, we report only the effects involving the factor MappingTransition (same as on trial n-1 vs. different).

Gener switch/repeat × MappingTransition × CSI ANOVAs revealed a significant main effect of MappingTransition for RTs, *F*(1,35) = 13.83, *p* < .001, ηp^2^ = .283; for errors it only approached significance, *F*(1,35) = 3.75, *p* = .061, ηp^2^ = .097. For RTs, this effect was qualified by a significant interaction between MappingTransition and switch/repeat, *F*(1,35) = 17.15, *p* < .001, ηp^2^ = .329, reflecting a large benefit of hearing each gender (voice) on the same side as on the previous trial (compared to swapping the sides of the two genders) for target gender repetitions and little/no such benefit for target gender switches. This was confirmed by separate ANOVAs (with factors MappingTransition and CSI) for target gender repetitions, where the main effect of MappingTransition was significant, *F*(1,35) = 56.53, *p* < .001, ηp^2^ = .618, and for gender switches, where this effect did not approach significance *F*(1,35) = 0.004, *p* = .951, ηp^2^ < .001.

Importantly, neither the 2-way interaction between MappingTransition and CSI, nor the 3-way interaction of these factors with switch/repeat, approached significance for either RTs or errors (all Fs < 1).

### Discussion

In Experiment 2 we asked whether the RISC effect found for gender-defined selection of voices presented non-spatially (diotically) would also be observed when voices have a randomly varying spatial dimension (ear). RT analyses revealed a significant RISC effect, whose magnitude (52%) was comparable to that observed in the gender session of Experiment 1 in the absence of the ear dimension (59%).

The results revealed, as in Experiment 1, a clear effect of the trial-to-trial repetition vs. change of gender-ear mappings on performance, which again manifested itself as a benefit for trials where each voice was presented on the same side as on the previous trial compared to trials where the voices swapped sides relative to trial n-1, although in the current experiment this benefit was significant only for repetitions of the relevant (gender) dimension. The pattern we observed is consistent with the effect reported by Koch and Lawo (2014) that the switch cost was larger when the ear where the target gender was presented remained the same as on the preceding trial – this is also the case in our experiments (see Figures 3 and 6).

As in the ear session of Experiment 1, the trial-to-trial transitions of cross-dimensional mappings did not interact significantly with the RISC effect. Thus, the presence of the salient, task-irrelevant, randomly changing, spatial dimension did not discourage (or detectably reduce the effectiveness of) preparatory gender-cued shifts of the auditory attention between concurrent voices. Finally, Experiment 2 confirmed the performance advantage (especially for the short CSI) for the picture cues revealed in Experiment 1 and reported previously (see Introduction). However, unlike in the ear session of Experiment 1, the type of cue did not significantly affect the switch cost.

## General Discussion

Over the last decade or so, one novel approach to the cocktail party problem has been the investigation of psychological processes underlying intentional (goal-related) switches of auditory attention between talkers by means of the voice-cueing paradigm (e.g., Koch et al., 2011). These studies and studies using other kinds of paradigm (e.g., Best et al., 2008) have revealed a substantial performance cost associated with shifting attention among voices, or their spatial locations. As already explained in the Introduction, the switch cost may arise from multiple sources, hence further measures are required to ascertain the contribution of top-down control. One such measure is the reduction in switch cost with preparation – the RISC effect. It indicates that components of the task-set (including types of attentional/perceptual selection) can be reconfigured in advance of the imperative stimulus. However, until recently the RISC effect has been elusive in cocktail party settings, prompting doubts regarding the generality of advance control processes posited by the task-set reconfiguration accounts (see Introduction).

### Preparatory Shifts of Spatial and Non-Spatial Attention in the Cocktail Party Setting

The primary objective of the current study was to apply our preparation-focused methodology to spatial auditory attention in the multitalker setting – where the evidence for spatial preparatory switches has been modest thus far. Our results from the experimental session in Experiment 1 where the target voice was cued spatially (by side/ear), revealed a significant reduction in switch cost with preparation for both RTs and errors. The RT RISC effect was of similar magnitude to the RISC effect documented in recent studies (Monsell et al., 2019; Strivens et al., 2024) for gender cueing. To our knowledge, this is the first conclusive demonstration that spatial auditory attention can be shifted between simultaneous voices in advance of their onset (in silence), and that such preparatory shifts benefit switching performance.

A further aim was to establish whether the RISC effect we found for gender-cued selection of diotically (non-spatially) presented voices generalises to dichotic presentation, and, in particular, examine the possibility that random changes in the location (side) of each voice may discourage preparation for a switch of the target speaker or reduce the effectiveness of such preparation. Indeed, this possibility is suggested by previous gender-cueing studies that did not find a reliable RISC effect presented voices dichotically. Experiment 2 showed that the RISC effect does generalise to dichotic presentation and is not materially affected by the presence of the salient, but task-irrelevant, spatial dimension. The magnitude of the RISC effect (52%) in Experiment 2 is in-between the RISC effects in Monsell et al. (40%) and in the gender session of Experiment 1 (59%). This and the outcomes from the ear session of Experiment 1, where the task-irrelevant gender (and therefore identity) of the talker varied randomly, show that preparation for a change in a non-spatial or spatial voice feature can be effective despite the uncorrelated salient task-irrelevant perceptual dimension.

The above results clearly demonstrate that the task-set reconfiguration account generalises to auditory attention shifts among simultaneous talkers. It also seems that the earlier failures or inconsistencies in finding the RISC effect in voice switching are not due to some fundamental limitation of preparatory retuning auditory attention from one voice to another. On the contrary, it seems that preparatory attentional shifts can be effective, reducing the switch cost by as much as 40–60%. Given the substantial RISC effects in the current experiments, but also in the recent studies by Monsell et al. (2019) and Strivens et al. (2024a), one is tempted to ask why the effect of preparation on the switch cost was elusive in earlier studies reviewed in the Introduction? Might the RISC effect be confined to a very specific set of experimental parameters? We think not. As already mentioned in the Introduction, Monsell et al. (2019) have shown that preparation reduces the switch cost to a comparable extent for: (a) highly familiar voices vs. voices not heard before the experiment, and (b) for simultaneous vs. staggered voices (one voice had an earlier onset) – and for the latter the RISC effect was not materially influenced by the order of the target voice and the non-target voice. Recently, Strivens et al. (2024a) found substantial and statistically indistinguishable RISC effects when voice compounds presented on the majority of the trials were response-incongruent (as in the current experiments) and when the voice compounds on the majority of the trials were response-congruent. The present experiments have revealed clear RISC effects both in the absence and in the presence of a salient, but task-irrelevant, perceptual dimension of voices. Thus, the RISC effect is present for a wide range of conditions/parameters in voice switching.

This being said, the available evidence points to three kinds of factor that do (or are likely to) influence the likelihood, effectiveness and detectability of the RISC effect in the cocktail party setting. First, as in conventional task switching studies (e.g., Monsell & Mizon, 2006) the probability of a switch has a substantial effect both on the target voice switch cost and on the RISC effect (Strivens et al., 2024a; Strivens et al., 2024b). In particular, Strivens et al.’s (2024a) found significant RISC effect when the probability of a voice switch was 0.25, but not when it was 0.75. However, the difference in switch probability between the recent studies that have vs. have not revealed a significant RISC effect has been much smaller (0.33 vs. 0.5) than in Strivens et al.’s (2024a), hence switch probability is unlikely to be the only factor that explains the discrepancies in the reported RISC effects. Second, all the studies that revealed a RISC effect used a single voice per gender. This especially relevant for gender-cued selection because it allows the participant to prepare for a specific voice – which was not possible in earlier studies that used multiple voices per gender. Further research will need to test the role of this factor systematically. Finally, the studies that reported a robust RISC effect had this effect as the primary topic of investigation, ensuring adequate to generous cell sizes (and therefore statistical power) for detecting the effect. Conversely, in at least some of the studies where a non-significant RISC effect was reported, the relevant analyses may have not been sufficiently sensitive for detecting the RISC effect.

### Effects of Interactions Between Spatial and Non-Spatial Dimensions

Another important aspect of our investigation concerns the interaction between the spatial (ear) and non-spatial (speaker/gender-related) dimensions of voices and their effect on the attention switch cost and its reduction with preparation. In Experiment 1 these effects were examined for a condition in which the target voice was defined (and cued) by its location (side/ear) and the speaker identity (and gender) was irrelevant. In Experiment 2 the relevant and irrelevant dimensions were reversed. Koch and Lawo (2014) proposed that the features of the two dimensions form episodic bindings (e.g. male-left), which subsequently result in extra benefits when the values on both dimensions are repeated relative to repeating only the value of the relevant dimension. A related account proposed by Best and colleagues (2008) is that the integration over non-spatial and spatial features of voices into auditory objects requires temporal continuity. In the current experiments, performance was better when the side (ear) where each voice was presented remained the same as on the preceding trial. Importantly, we found no indication that the trial-to-trial dynamics of such mappings interacted with the RISC effect.

The cross-dimension links/bindings formed on the preceding trial seemed to benefit performance less when the target value on the task-relevant dimension switched than when it repeated (significantly so in Experiment 2). This may suggest a further process at play – possibly a form of *perceptual priming*. When the target feature on the relevant dimension (e.g., ear) is repeated and the gender-ear mapping (e.g., female-left, male-right) is the same as on trial n-1, the participant hears in the cued ear (e.g., left) the same voice/gender as on the previous trial (female). Perceptual processing of the same fundamental frequency (and other non-spatial characteristics specific to this voice) as on the preceding trial likely results in priming and therefore a performance benefit. When the target feature on the relevant dimension switches (e.g., from left to right) and the gender-ear mapping (female-left, male-right) is the same as on trial n-1, the participant hears in the newly-cued ear (right) a different voice from trial n-1 (male voice) – so there is no benefit of encoding the same voice. When the target feature switches (e.g., from left to right) and the gender-ear mapping from trial n-1 (e.g., female-left, male-right) changes (female-right, male-left), the participant hears in the newly cued tear (right) the same voice they listened to on trial n-1 (female), leading to a benefit of processing the same fundamental frequency and vocal tract length – but swapping the gender-ear mapping is likely detrimental for performance due to the binding (male-right) retrieving the male voice when attending to the right side (see above). Thus, for target switches perceptual priming and binding of features from across dimensions may have opposing effects on performance where they may (partially) cancel out. For target repetition trials, these factors have co-directional effects, hence the large benefit from having the same gender-ear mappings as on trial n-1 is likely may be due not only to binding but also to perceptual priming.

### Effects of Type of Voice Cue

As explained in the Introduction, the need for (at least) two cues for each gender (or ear) was dictated by the need to minimise the confounding effect of immediate cue repetitions on the target voice switch cost and its reduction with preparation. This also allowed for the comparison of different types of visual cue: word and picture. In both experiments, for both selection criteria (gender and ear), and for both performance measures (RT and errors), picture cues resulted in better performance than word cues, fully supporting the initial report by Monsell et al. (2019). The advantage of picture cues over verbal cues in voice-cueing experiments is the reverse of the effect previously reported in task-switching studies (e.g., Lavric et al., 2008; Monsell & Mizon, 2006, see Introduction). We propose that the handicap of word cues in the context of voice cueing is that they inadvertently elicit covert phonological processing which interferes with the perceptual encoding of speech. This interpretation is supported by the current finding in both experiments of a steep reduction of the advantage of the picture cues as the CSI increased and the disappearance of this advantage for the CSI = 1400 ms in the ear session of Experiment 1 and in Experiment 2.

The effect of the type of cue on the switch cost is less clear. In the present study, the error switch cost was smaller for picture cues for spatial selection (ear session, Experiment 1), but not for the gender-based selection in the two experiments. In Monsell et al. (2019) gender-cueing study, the RT switch cost was smaller for the picture cues when the two voices were familiar and the picture cues were the photographs of participants’ own parents – it was not found in the following experiment where the picture cues were the silhouettes also used in the current study. Finally, neither Monsell et al.’s (2019) experiments, nor the present experiments, revealed a notable influence of cue type on the RISC effect.

### Conclusions and Remaining Questions

The current experiments document a pattern of performance – the RISC effect – indicative of preparatory shifts of attention during the (silent) cue-stimulus interval that precedes the onset of two simultaneous voices. To the best of our knowledge, this is the first conclusive demonstration of a RISC effect when the target voice is selected spatially. We also replicate the effect of preparation on the switch cost we previously reported for gender-based selection of voices presented non-spatially (centrally) and extend it to lateralised (dichotic presentation). These results show that theories based on advance endogenous task-set control (e.g., Monsell, 2003; 2015) can be extended to shifting attention between simultaneous voices.

However, there are important questions that remain. First, although the present and previous experiments (Monsell et al., 2019; Strivens et al., 2024a) have provided useful insights on experimental variables that do or do not seem to affect preparatory shifts of attention, further work is required to ascertain all the factors that encourage the advance control of auditory attention and maximise its measurement. At least one such factor has been identified – the probability of a voice switch. Second, preparation reduces, but far from eliminates, the switch cost – which suggests at least some sources of the switch cost that are immune to preparation. These may include inertia of the attentional settings from the preceding trial and/or other processes that will need to be examined by future studies.

Another key aspect of our results concerns the relation between the task-relevant voice dimension (attribute) and the task-irrelevant dimension. Our analyses show for both spatial and non-spatial selection of a voice that maintaining the mapping/configuration of spatial and non-spatial features (e.g. male voice on the left, female voice on the right) from one trial to the next benefits performance. This suggests rapidly formed cross-dimensional feature bindings and potentially emerging auditory objects. But – neither the presence of an irrelevant dimension, nor the trial-to-trial dynamics in cross-dimension mappings seem to have a detectable influence on the RISC effect, suggesting that preparatory shifts operate effectively within a single (relevant) dimension. It can be argued that the latter may be due (at least in part) to the design of our experiments where the mapping between features on the two dimensions changes frequently and unpredictably – which discourages rapid integration over dimensions. It would be interesting to further explore preparatory tuning of attention in conditions which (sometimes) encourage stronger cross-dimension integration.

Finally, our results show that pictures are more effective at cueing the target voice than words, especially when the presentation of the cue is closer in time to the onset of voices. This suggests that phonological processes triggered by verbal cues (even in the visual modality) may interfere with selective listening. This finding has obvious practical implications for cueing speakers in multitalker settings, but further research is required to confirm the generality of this result and ascertain its underlying mechanism.

## Data Accessibility Statement

Materials, scripts and data and are available at https://osf.io/g72mk/.

## Footnotes

Given that each participant heard only one man and only one woman, it seems likely that they learned to tune their auditory attention to specific voices rather than the voice’s gender (see above).

Given the variability in the exact shape of cues, we provide the size of the smallest rectangle that comprises each cue.

Pilot work in our laboratory revealed that if 100% of trials were incongruent some participants reported using this strategy.

As already mentioned, although in this session the target voice was cued by gender, it is likely that participants learned to tune their auditory attention to the specific non-spatial characteristics of the individual speaker.

Because in within-subjects designs the variability within each condition is uninformative with regard to the variability of the contrast between conditions, we provide in figures the variability statistic (Standard Error) for the difference between conditions – the switch cost.

The main effect of the MappingTransition factor in our analysis is statistically equivalent to the 2-way interaction in Koch and Lawo (2014) between target gender switch/repetition and the factor that coded whether the ear in which the target gender was presented switched or was repeated.
